# Advances in the treatment of hereditary transthyretin amyloidosis: A review

**DOI:** 10.1002/brb3.1371

**Published:** 2019-08-01

**Authors:** Morie A. Gertz, Michelle L. Mauermann, Martha Grogan, Teresa Coelho

**Affiliations:** ^1^ Mayo Clinic Rochester Minnesota; ^2^ Centro Hospitalar do Porto Hospital de Santo António Porto Portugal

**Keywords:** amyloid, hATTR, inotersen, patisiran, transthyretin amyloidosis

## Abstract

**Introduction:**

Amyloid transthyretin amyloidosis (ATTR) is a progressive and often fatal disease caused by the buildup of mutated (hereditary ATTR [hATTR]; also known as ATTR variant [ATTRv]) or normal transthyretin (wild‐type ATTR) throughout the body. Two new therapies—inotersen, an antisense oligonucleotide therapy, and patisiran, an RNA interference therapy—received marketing authorization and represent a significant advance in the treatment of amyloidosis. Herein, we describe the clinical presentation of ATTR, commonly used procedures in its diagnosis, and current treatment landscape for ATTR, with a focus on hATTR.

**Methods:**

A PubMed search from 2008 to September 2018 was conducted to review the literature on ATTR.

**Results:**

Until recently, there have been few treatment options for polyneuropathy of hATTR. Inotersen and patisiran substantially reduce the amyloidogenic precursor protein transthyretin and have demonstrated efficacy in patients with early‐ and late‐stage disease and in slowing or improving neuropathy progression. In contrast, established therapies, such as liver transplantation, typically reserved for patients with early‐stage disease, and tafamidis, indicated for the treatment of early‐stage disease in Europe, or diflunisal, a nonsteroidal anti‐inflammatory drug that is used off‐label, are associated with side effects and/or unclear efficacy in certain patient populations. Thus, inotersen and patisiran are positioned to be the preferred therapeutic modalities.

**Conclusions:**

Important differences between inotersen and patisiran, including formulation, dosing, requirements for premedications, and safety monitoring, require an understanding and knowledge of each treatment for informed decision making.

## 1 INTRODUCTION

Amyloid transthyretin (TTR) amyloidosis (ATTR amyloidosis) is a rare, progressive, and fatal disease caused by the buildup of amyloid fibrils in organs and tissues (Connors et al., 2016; Koike et al., 2016; Koike, Yasuda, et al., 2018). It can be caused by the buildup of mutated TTR, referred to as hereditary ATTR (hATTR) or ATTR variant (ATTRv), or by the buildup of normal TTR, referred to as wild‐type ATTR (wtATTR) (Connors et al., 2016; Koike et al., 2016; Koike, Yasuda, et al., 2018). hATTR is transmitted in an autosomal dominant manner, and more than 130 *TTR* gene mutations have been identified thus far (Rowczenio & Wechalekar, 2018). The mechanism by which wild‐type *TTR* becomes amyloidogenic is poorly understood. Until recently, therapeutic options have been limited for patients with hATTR. This review describes the clinical presentation and commonly used diagnostic procedures and provides an overview of the current treatment landscape for ATTR, with a focus on hATTR.

## 2 METHODS

A PubMed search from 2008 to September 2018, with the search terms “amyloid,” “amyloidosis,” “familial amyloid cardiomyopathy,” “familial amyloid polyneuropathy,” “inotersen,” “patisiran,” “tafamidis,” “diflunisal,” “liver transplant,” “doxycycline,” “AG10,” “PRX004,” “anti‐SAP,” “TTRsc02,” “epigallo‐catechin‐3‐gallate,” “SOM0226,” “tolcapone,” “NI‐301,” “RT24,” “NPT088,” “NPT289,” “LUNAR‐TTR,” “PTI‐110,” and “tauroursodeoxycholic acid,” limited to English‐only publications, was conducted. Reference lists were also reviewed to identify key publications.

## 3 DISCUSSION/OBSERVATION

### 3.1 Clinical presentation

Hereditary ATTR manifests as sensorimotor neuropathy, autonomic neuropathy, cardiomyopathy, and nephropathy, whereas wtATTR mainly affects the heart, especially in men ˃60 years old (Table 1) (Carr et al., 2016; Conceicao et al., 2016; Connors et al., 2016; Maurer et al., 2016). Of note, manifestations consistent with peripheral and autonomic neuropathy involvement have been observed in patients with wtATTR (Connors et al., 2016; Maurer et al., 2016). A classic feature of hATTR is length‐dependent peripheral sensorimotor neuropathy (Cappellari et al., 2011; Carr et al., 2016). Symptoms typically progress in a distal to proximal direction with feet affected first followed by upper limb involvement (Carr et al., 2016). As the disease progresses, patients experience increasing lower limb muscle weakness, walking difficulty, imbalance, and sensory loss (Carr et al., 2016; Coelho et al., 2017; Maurer et al., 2016). Neurologic symptoms and the pattern of progression of neurologic manifestations may vary according to mutation type (Cappellari et al., 2011; Carr et al., 2016). Recognizing hATTR as a cause of polyneuropathy may be challenging because of its similarity to more common causes of neuropathy. Symptoms that may help to distinguish hATTR from other causes of neuropathy include neuropathic pain (often described as lightning pain), autonomic dysfunction, absence of ataxia, small fiber sensory loss above the wrist, and weakness in the upper limbs, and are more common in hATTR (Lozeron et al., 2018). Autonomic neuropathy can manifest as orthostatic hypotension and/or sexual dysfunction but may not be reported (Carr et al., 2016; Koike, Nakamura, et al., 2018; Lozeron et al., 2018; Maurer et al., 2016; Wixner, Tornblom, Karling, Anan, & Lindberg, 2018). Therefore, patients presenting with progressive length‐dependent neuropathy of unknown origin, particularly those with concomitant autonomic dysfunction, should be tested for ATTR. Additional symptoms include progressive cardiomyopathy and gastrointestinal disturbances. Progressive cardiomyopathy results in a rapid decline in cardiac functional capacity (Castano et al., 2016; Ruberg et al., 2012). Patients may experience rhythm disturbances, dyspnea, syncope, and palpitations (Maurer et al., 2016). Gastrointestinal disturbances include gastroparesis leading to nausea and vomiting, alternating diarrhea and constipation, and unintentional weight loss (Coelho, Maurer, & Suhr, 2013; Maurer et al., 2016; Wixner et al., 2018).

**Table 1:** Signs, symptoms, and “red‐flag” manifestations^a^ of ATTR amyloidosis (Castano et al., 2016; Coelho et al., 2008; Conceicao et al., 2016; Donnelly & Hanna, 2017; Galat et al., 2016; Geller et al., 2017; Gertz, 2017; Ruberg & Berk, 2012; Sperry et al., 2018)

	Signs, symptoms, and manifestations^b^
Neuropathy	Progressive symmetric peripheral sensorimotor neuropathy (plus ≥ 1 other “red‐flag” manifestation) is suggestive of hATTR^a^
Bilateral carpal tunnel syndrome	Bilateral carpal tunnel syndrome (especially if family history)^a^
Autonomic neuropathy	Orthostatic hypotension^a^ Erectile dysfunction^a^ Recurrent urinary tract infection (due to urinary retention) Sexual dysfunction Sweating abnormalities
Cardiovascular manifestations	Irregular heartbeat (atrial fibrillation most common)^a^ Conduction blocks (including bundle branch blocks)^a^ Congestive heart failure (including shortness of breath, generalized fatigue, and peripheral edema) Ventricular wall thickening with preserved ejection fraction and absence of left ventricular dilation^a^ Cardiomyopathy Mild regurgitation
Gastrointestinal manifestations	Nausea and vomiting Early satiety Chronic diarrhea^a^ Severe constipation^a^ Diarrhea/constipation^a^ Unintentional weight loss^a^
Nephropathy	Albuminuria^a^ Mild azotemia^a^ Protein in urine Renal failure
Ocular manifestations	Dark floaters^a^ Glaucoma Abnormal blood vessels in eye Pupillary abnormalities
Other	Lumbar spinal stenosis^a^ Spontaneous distal biceps tendon rupture^a^

Abbreviations: ATTR, amyloid transthyretin; hATTR, hereditary amyloid transthyretin amyloidosis.

a Red‐flag manifestations.

b Central nervous system symptoms can occur with certain *TTR* mutations but are not a common manifestation.

An early, potential red‐flag manifestation of ATTR amyloidosis is bilateral carpal tunnel syndrome (Carr et al., 2016; Nakagawa et al., 2016). Bilateral carpal tunnel syndrome in combination with progressive symmetric sensorimotor neuropathy and/or cardiomyopathy should raise suspicion of ATTR (Carr et al., 2016; Conceicao et al., 2016; Nakagawa et al., 2016; Sperry et al., 2018).

### 3.2 Assessment and diagnosis of ATTR

**Figure 1:**
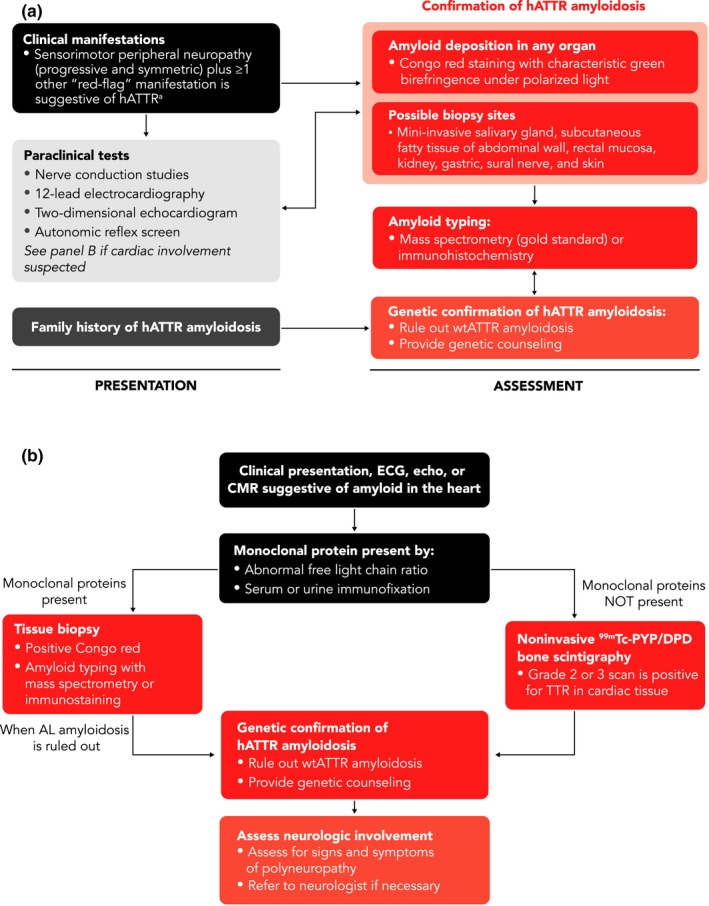
Diagnostic workup for polyneuropathy (a) and cardiomyopathy (b) when ATTR amyloidosis is suspected (Carvalho et al., 2015; Castano et al., 2016; Coelho et al., 2008; Conceicao et al., 2016; Donnelly & Hanna, 2017; Galat et al., 2016; Geller, Singh, Alexander, Mirto, & Falk, 2017; Gertz, 2017, 2018; Gillmore et al., 2016; Nativi‐Nicolau & Maurer, 2018; Ruberg & Berk, 2012; Sperry et al., 2018). ^99m^Tc, technetium‐99m; AL, amyloid light chain; ATTR, amyloid transthyretin; CMR, cardiac magnetic resonance imaging; DPD, 3,3‐diphosphono‐1,2‐propanodicarboxylic acid; ECG, electrocardiogram; echo, echocardiogram; hATTR, hereditary amyloid transthyretin; MRI, magnetic resonance imaging; PYP, pyrophosphate; TTR, transthyretin; wtATTR, wild‐type amyloid transthyretin. Panel A: ^a^“Red‐flag” manifestations include progressive symmetric peripheral sensorimotor neuropathy plus ≥ 1 of the following: bilateral carpal tunnel syndrome (especially family history), orthostatic hypotension, erectile dysfunction, irregular heartbeat (especially atrial fibrillation), conduction blocks (including bundle branch blocks), ventricular wall thickening with preserved ejection fraction and absence of left ventricular dilation, chronic diarrhea, severe constipation, diarrhea/constipation, unintentional weight loss, albuminuria, mild azotemia, dark floaters, lumbar spinal stenosis, and/or spontaneous distal biceps tendon rupture. Modified with permission from Gertz (2017)

In patients with signs, symptoms, or manifestations suggestive of amyloidosis, diagnostic and genetic testing are of utmost importance. Neurologic and cardiac symptoms can be evaluated with numerous tests and procedures, including tissue biopsy, Congo red staining to confirm the presence of amyloid, and mass spectrometry or immunohistochemistry to confirm the type of amyloid (Figure 1a,b) (Benson et al., 2011; Carvalho, Rocha, & Lobato, 2015; Coelho, Maurer, et al., 2013; Gilbertson et al., 2015; Linke, Oos, Wiegel, & Nathrath, 2006; Sperry et al., 2018). Genetic testing is required to differentiate wtATTR from hATTR and to allow for the detection of specific *TTR* gene mutations, which may help predict the clinical course of disease (Coelho, Maurer, et al., 2013). In patients with a family history, it may be appropriate to proceed directly to genetic testing.

Myocardial radiotracer uptake on bone scintigraphy is an alternative to tissue diagnosis for patients with cardiac ATTR (Gillmore et al., 2016). Myocardial uptake of bone scintigraphy agents (e.g., ^99m^technetium [Tc]‐pyrophosphate scintigraphy and ^99m^Tc‐3,3‐diphosphono‐1,2‐propanodicarboxylic acid) is sensitive and specific for the diagnosis of TTR cardiac amyloid, if results of screening tests for light chain amyloid (serum and urine electrophoresis with immunofixation and serum free light chains) are negative (Bokhari et al., 2018; Gertz et al., 2015; Gillmore et al., 2016; Nativi‐Nicolau & Maurer, 2018). The use of bone scintigraphy is more sensitive than echocardiography in detecting early cardiac ATTR. Patients with cardiac amyloid detected via scintigraphy should also be evaluated for neuropathy because patients with hATTR often have a mixed phenotype. This will ensure appropriate treatment.

### 3.3 Therapeutic landscape for ATTR

#### 3.3.1 Liver transplantation

Liver transplantation is a standard treatment option for hATTR because replacing the primary source of mutant TTR substantially reduces its production. The efficacy of liver transplantation has been demonstrated in studies showing improvement in sensory and motor impairment (Okumura et al., 2016), as well as improvement in long‐term overall survival (Yamashita et al., 2012). The 10‐year posttransplantation survival rate exceeds 70% for some patients with hATTR (Suhr, Larsson, Ericzon, & Wilczek, 2016). Despite reports of successful outcomes with liver transplantation, progression of peripheral and autonomic neuropathy and cardiomyopathy have occurred following liver transplantation (Banerjee et al., 2017). Additionally, numerous factors, such as genotype and pretransplantation (e.g., modified body mass index [mBMI]) characteristics, have been shown to affect posttransplantation survival (Banerjee et al., 2017; Suhr et al., 2016). The 10‐year survival rate is as low as 23% for patients with Ser50Arg hATTR and as high as 85% for patients with Val71Ala hATTR (Suhr et al., 2016). In multivariate analysis, the presence of Val30Met hATTR is associated with significantly reduced mortality (Ericzon et al., 2015). Additional factors to consider when selecting patients for transplantation are availability of donor liver/graft, patient fitness, the need for long‐term immunosuppression, and posttransplantation complications.

#### 3.3.2 Transthyretin stabilizers

**Figure 2:**
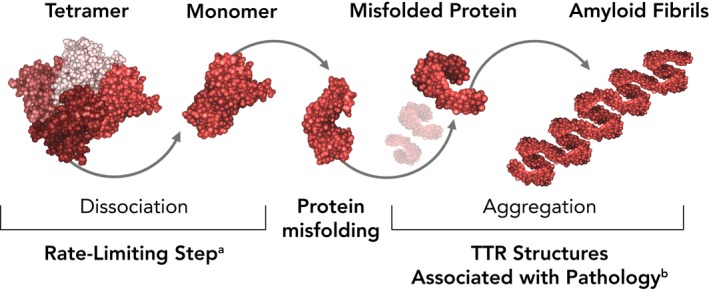
Mechanism of amyloid formation. ^a^Rate‐limiting step involves dissociation of tetrameric TTR to a pair of dimeric TTRs, which rapidly progresses to monomeric TTR. ^b^Misfolded protein can form a variety of toxic intermediates, including amyloid fibrils (shown here); small oligomers; and amorphous aggregates. TTR, transthyretin. Modified with permission from Bulawa et al. (2012)

In hATTR, mutations are thought to weaken interactions between the normally tetrameric TTR, causing dissociation into monomeric TTR, a species prone to misfolding and aggregation (Figure 2) (Bulawa et al., 2012; Saldano, Zanotti, Parisi, & Fernandez‐Alberti, 2017). The rate‐limiting step in fibril formation is TTR tetramer dissociation to folded dimers. Therefore, preventing TTR tetramer dissociation is one approach to preventing amyloid fibril formation and is the mechanism of action of the TTR stabilizers tafamidis and diflunisal (Bulawa et al., 2012; Sekijima, Dendle, & Kelly, 2006).

##### Tafamidis

Tafamidis, a kinetic TTR stabilizer, binds to the unoccupied thyroxine binding sites of tetrameric TTR, preventing the amyloidogenic cascade (Bulawa et al., 2012; Pfizer Ltd, 2016). It is approved in Europe for the treatment of ATTR amyloidosis in adults with stage 1 symptomatic polyneuropathy (Pfizer Ltd, 2016) (graded on a scale from 1 [lowest disease burden] to 3 [greatest disease burden]) (Coelho et al., 2017). It is approved in several other countries (e.g., Japan, Brazil, Mexico), but it is not approved in the United States for the treatment of polyneuropathy of ATTR amyloidosis.

The efficacy and safety of tafamidis have been demonstrated in patients with early‐stage hATTR polyneuropathy and most recently in patients with hATTR cardiomyopathy; however, the drug's efficacy in mid‐ to late‐stage hATTR and across a broad range of hATTR genotypes is mixed. Two studies in patients with early‐stage hATTR polyneuropathy—Fx‐005 (clinicaltrials.gov identifier: NCT00409175) and Fx‐006 (clinicaltrials.gov identifier: NCT00791492)—demonstrated less neurologic deterioration with tafamidis 20 mg once daily compared with placebo but did not reach statistical significance (Coelho, Maia, et al., 2013; Coelho et al., 2012). The efficacy and adverse event profile observed in these studies led to the approval of tafamidis in the European Union in 2011 (Pfizer Ltd, 2016). Sustained activity and tolerability of tafamidis for up to 6 years have been reported (clinicaltrials.gov identifier: NCT00925002) (Barroso et al., 2017); however, outcomes are mixed for patients with mid‐ to late‐stage Val30Met hATTR and patients with any‐stage non‐Val30Met hATTR. Neuropathy progression and increasing disability in patients with late‐stage Val30Met hATTR (Lozeron et al., 2013) and worsening in neurologic function in patients with non‐Val30Met hATTR have been demonstrated (Merlini et al., 2013). In addition to neuropathy progression in patients with non‐Val30Met hATTR, Cortese et al. (2016) reported worsening in autonomic function in patients with baseline dysautonomia and worsening in cardiac function in patients with baseline cardiac disease. Furthermore, albeit based on a single report, Cortese et al reported approximately one‐third of patients without cardiac involvement at baseline developed cardiac disease during tafamidis treatment. In general, tafamidis was well tolerated. The mixed outcomes in patients with mid‐ and late‐stage Val30Met hATTR or with any‐stage non‐Val30Met hATTR and the limited impact on polyneuropathy in general underscore the need to identify patients who do not respond to tafamidis so that other treatments might be considered.

Recently, the efficacy and safety of tafamidis were evaluated in the treatment of ATTR cardiomyopathy. A double‐blind, placebo‐controlled, phase 3 study involving patients with wtATTR or hATTR compared the efficacy and safety of tafamidis 20 mg or 80 mg once daily and placebo (ATTR‐ACT; clinicaltrials.gov identifier: NCT01994889) (Maurer et al., 2018). After 30 months of follow‐up, a significant reduction in all‐cause mortality and cardiovascular‐related hospitalization, assessed hierarchically, was observed for tafamidis (20 and 80 mg once daily pooled) compared with placebo. Tafamidis reduced the rate of all‐cause mortality by 30% and cardiovascular‐related hospitalizations by 32%. Although benefit was observed with tafamidis, prespecified subgroup analysis showed that in patients with New York Heart Association class III disease, cardiovascular‐related hospitalizations were higher among patients receiving tafamidis than among those receiving placebo. Additionally, all‐cause mortality and cardiovascular hospitalization did not reach statistical significance in patients with hATTR. Patients receiving tafamidis experienced a significant reduction in the decline of functional capacity (assessed by the 6‐min walk test) and quality of life (QOL; assessed by the Kansas City Cardiomyopathy Questionnaire–Overall Summary score) than patients receiving placebo.

Findings from this study provided data for the approval of tafamidis meglumine (Vyndaqel, Pfizer, Inc.) and tafamidis (Vyndamax, Pfizer, Inc.) in the treatment of cardiomyopathy of wtATTR or hATTR in adults to reduce cardiovascular mortality and cardiovascular‐related hospitalization (Pfizer, 2019).

##### Diflunisal

Diflunisal, a nonsteroidal anti‐inflammatory drug (NSAID), has TTR‐stabilizing properties and has been used off‐label for the treatment of hATTR. In a randomized, placebo‐controlled, double‐blind, investigator‐initiated study (clinicaltrials.gov identifier: NCT00294671), the difference in Neuropathy Impairment Score + 7 neurophysiologic tests composite score (NIS + 7) between diflunisal 250 mg twice daily and placebo at 2 years indicated decreased neuropathy progression (Berk et al., 2013). Additionally, physical and mental scores on the 36‐Item Short‐Form Health Survey indicated improvement in QOL.

Despite the efficacy of diflunisal, adverse events associated with NSAIDs—specifically gastrointestinal, renal, cardiac, and blood‐related events—are a concern for patients with hATTR and may preclude use of diflunisal in specific patients. NSAID‐related adverse events, including acute renal failure (Azorin, Cabib, & Campistol, 2017), deterioration in renal function, and thrombocytopenia (Sekijima, Tojo, Morita, Koyama, & Ikeda, 2015), have been shown in 2 recent studies conducted in Spain and Japan.

Collectively, tafamidis and diflunisal have demonstrated benefit in the treatment of hATTR, but their benefit may be limited by potential variable efficacy in specific patient populations and by an increased potential for adverse events (diflunisal).

#### 3.3.3 Transthyretin protein knockdown (reduction) agents (gene silencing)

Inotersen and patisiran recently received marketing authorization for the treatment of neuropathy. These agents substantially reduce TTR protein levels by degrading *TTR* mRNA, specifically degradation of mRNA via nuclear RNaseH1 with inotersen or cytoplasmic RNA‐induced silencing complex with patisiran (Ackermann et al., 2016; Adams, Gonzalez‐Duarte, O'Riordan, Yang, Ueda, et al., 2018). Both mechanisms of action differ from liver transplantation in that both mutated and wild‐type TTR production are targeted. Reductions in mutated and wild‐type TTR delay or halt progression of existing neurologic manifestations, and reductions in wild‐type TTR may prevent the development of new manifestations. Both inotersen and patisiran have demonstrated efficacy in patients with early‐ and late‐stage neurologic disease; however, they differ in terms of formulation, dosing, premedication requirements, and safety monitoring (Table 2).

**Table 2:** Main properties of TTR gene knockdown therapies (Ackermann et al., 2016; Adams, Gonzalez‐Duarte, O'Riordan, Yang, Ueda, et al., 2018; Akcea Therapeutics, Inc., 2018a; Alnylam Netherlands B.V., 2018; Alnylam Pharmaceuticals Inc., 2018; Ionis USA Ltd, 2018)

	Inotersen	Patisiran
Indication	Polyneuropathy of hATTR in adults (United States) Stage 1 or 2 polyneuropathy in adults with hATTR (European Union)	Polyneuropathy of hATTR in adults (United States) Stage 1 or 2 polyneuropathy in adults with hATTR (European Union)
Therapeutic dose	284 mg	0.3 mg/kg For patients weighing ≥100 kg, adjust dose to 30 mg once every 3 weeks
Administration	Once weekly	Every 3 weeks
Route of administration	Subcutaneous	Intravenous
Premedication	Not required	Intravenous corticosteroid (e.g., dexamethasone 10 mg or equivalent) Oral acetaminophen (500 mg) Intravenous H1 blocker (e.g., diphenhydramine 50 mg or equivalent) Intravenous H2 blocker (e.g., ranitidine 50 mg or equivalent)
Precautions	Vitamin A supplementation at a dose consistent with the recommended daily allowance is warranted	Vitamin A supplementation at a dose consistent with the recommended daily allowance is warranted
Mechanism of action	Antisense oligonucleotide inhibitor that degrades mRNA via nuclear RNaseH1 Inhibits human TTR production by selectively binding to *TTR* mRNA Degrades both mutant and wild‐type (normal) *TTR* mRNA	Small interfering RNA that degrades TTR via cytoplasmic RNA‐induced silencing complex Inhibits human TTR production by selectively binding to *TTR* mRNA Degrades both mutant and wild‐type (normal) *TTR* mRNA
TTR reduction	79% (median nadir [predose] weeks 13–65)	81% (median reduction [postdose] over 18 months)
Safety monitoring^a^	Thrombocytopenia o Monitor platelet count weekly during treatment if values are ≥100 × 10^9^/L o Stop treatment if platelet count is ≥75 to ˂100 × 10^9^/L; monitor platelet count weekly until values resume to ˃100 × 10^9^/L o Stop treatment if platelet count is ≥50 to ˂75 × 10^9^/L; monitor platelet count twice weekly until 3 successive values resume to ˃75 × 10^9^/L; once values resume, monitor weekly o Stop treatment if platelet count is ≥25 to ˂50 × 10^9^/L; monitor platelet count twice weekly until 3 successive values resume to ˃75 × 10^9^/L; once values resume, monitor weekly. More frequent monitoring is warranted if risk factors for bleeding are present; corticosteroids (glucocorticoids) are strongly recommended, and discontinuation of antiplatelets/anticoagulants should be considered o Stop treatment if platelet count is ˂25 × 10^9^/L; monitor platelet count daily until 2 successive values resume to ˃25 × 10^9^/L; once values resume, monitor twice weekly until values resume to ˃75 × 10^9^/L; once values resume, monitor weekly. More frequent monitoring is warranted if risk factors for bleeding are present; corticosteroids (glucocorticoids) are strongly recommended, and discontinuation of antiplatelets/anticoagulants should be considered Glomerulonephritis/renal function decline o Monitor serum creatinine, eGFR, urinalysis, and UPCR every 2 weeks during treatment o Withhold inotersen if UPCR is ≥1,000 mg/g, or if eGFR is ˂45 ml/min/1.73 m^2^; dosing can resume once eGFR increases to ≥45 ml/min/1.73 m^2^, UPCR decreases to ˂1,000 mg/g, or the underlying cause of the decline in renal function is corrected o If UPCR is ≥2,000 mg/g, the presence of acute glomerulonephritis should be investigated; discontinue inotersen if acute glomerulonephritis is confirmed Liver function o Monitor ALT, AST, and total bilirubin every 4 months	Not required

Abbreviations: ALT, alanine aminotransferase; AST, aspartate aminotransferase; eGFR, estimated glomerular filtration rate; hATTR, hereditary amyloid transthyretin amyloidosis; TTR, transthyretin; UPCR, urine protein‐to‐creatinine ratio.

a Based on US prescribing information. The reader should refer to country‐specific monitoring and treatment recommendations.

##### Inotersen

Inotersen, a once‐weekly subcutaneously administered antisense oligonucleotide therapy, is approved in the United States for the treatment of the polyneuropathy of hATTR, and in Europe and Canada for the treatment of stage 1 or 2 polyneuropathy in adult patients with hATTR (Akcea Therapeutics, Inc., 2018a, 2018b; Ionis USA Ltd, 2018). Initial preclinical and clinical studies demonstrated sustained reductions in *TTR* mRNA and protein levels (Ackermann et al., 2016), and on the basis of these studies the phase 3, randomized, double‐blind, placebo‐controlled study NEURO‐TTR was initiated (clinicaltrials.gov identifier: NCT01737398) (Benson et al., 2018).

In NEURO‐TTR, 173 patients with hATTR polyneuropathy were randomly assigned 2:1 to receive subcutaneous inotersen 300 mg once weekly (*n* = 113; *n* = 112 treated) or placebo (*n* = 60) stratified by mutation status, disease stage, and prior stabilizer treatment (Benson et al., 2018). Baseline demographic and disease characteristics were generally balanced across both treatment groups; however, patients receiving inotersen had a longer mean duration of hATTR polyneuropathy from diagnosis compared with patients receiving placebo (mean, 42 vs. 39 months); this was particularly evident among patients with stage 2 hATTR (mean, 41 vs. 25 months) (Benson et al., 2018; Gertz et al., 2018). Additionally, a greater proportion of patients receiving inotersen than placebo had cardiomyopathy (67% vs. 55%) and had a longer duration from the onset of cardiomyopathy symptoms (mean, 48 vs. 34 months) (Benson et al., 2018; Gertz et al., 2018).

In NEURO‐TTR, patients treated with inotersen showed substantial reductions in TTR levels that were sustained over time (Benson et al., 2018; Dyck et al., 2018). Inotersen treatment resulted in a median TTR reduction from baseline (measured predose) of 75%–79% between months 3 and 15 (Dyck et al., 2018). Compared with placebo, patients receiving inotersen experienced a statistically significant improvement in modified NIS + 7 (mNIS + 7) and Norfolk Quality of Life–Diabetic Neuropathy questionnaire total score (Norfolk QOL‐DN) (co‐primary end points), as early as 8 months for mNIS + 7 (*p* ˂ .001) and Norfolk QOL‐DN (*p* = .03), with effects sustained at 15 months (both *p* ˂ .001) (Benson et al., 2018). Similar effects were observed for both mNIS + 7 and Norfolk QOL‐DN across all patient subgroups, based on study stratification factors, indicating clinical benefit across a broad spectrum of important disease characteristics (Benson et al., 2018). Improvement in neuropathy‐related QOL, as indicated by the reduction in Norfolk QOL‐DN scores, was corroborated by significant improvement in the physical component summary score of 36‐Item Short‐Form Health Survey at 15 months for inotersen compared with placebo (Benson et al., 2018).

Significant improvements from baseline to 15 months in favor of inotersen were observed in the subset of patients with both polyneuropathy and cardiomyopathy for the co‐primary end points, mNIS + 7 (*p* < .001) and Norfolk QOL‐DN (*p* = .04) (Benson et al., 2018).

Common adverse events, defined as adverse events occurring in ˃10% and twice as frequently in patients receiving inotersen than placebo, included nausea, pyrexia, chills, vomiting, anemia, thrombocytopenia, and lowered platelet counts (Benson et al., 2018). The rate of injection‐site reactions was 1.1% of all injections in patients who received inotersen. Most (97%) were mild in severity, and no patient discontinued because of injection‐site reactions. Five (4.5%) deaths occurred, and all were in inotersen‐treated patients; however, all but one were because of disease progression or underlying disease. A single death occurred because of fatal intracranial hemorrhage associated with serious thrombocytopenia in a patient whose platelets had not been monitored, as the event occurred before routine monitoring was implemented; similar events did not occur after monitoring was implemented (Benson et al., 2018). Safety concerns include thrombocytopenia and glomerulonephritis, which were monitorable and manageable following implementation of more frequent platelet (weekly) and renal (every 2–3 weeks) monitoring in the NEURO‐TTR study (Benson et al., 2018). In order to manage and minimize the potential risk of serious bleeding and glomerulonephritis, inotersen is available through a restricted distribution program under a Risk Evaluation and Mitigation Strategy (Akcea Therapeutics, Inc., 2018a). Enhanced monitoring for thrombocytopenia, glomerulonephritis/renal function, and liver function is recommended (Table 2) (Akcea Therapeutics, Inc., 2018a; Ionis USA Ltd, 2018).

The sustained benefit and tolerability of inotersen were demonstrated in the ongoing phase 3 open‐label extension study of NEURO‐TTR (clinicaltrials.gov identifier: NCT02175004) (Brannagan et al., 2018). In NEURO‐TTR, 81% of patients completed the 15‐month treatment period and ˃95% enrolled in the open‐label extension study (Benson et al., 2018). Extended dosing with inotersen, reaching 5.2 years for the longest time any patient received treatment, demonstrated continued slowing or improvement of neuropathy progression after 2 years of follow‐up (Brannagan et al., 2018). Additionally, initiation of inotersen in the extension study in patients who previously received placebo resulted in disease stabilization (Brannagan et al., 2018). Although patients who previously received placebo experienced rapid benefit, the delay in receiving inotersen resulted in less improvement in mNIS + 7 and Norfolk QOL‐DN than those of patients who started inotersen earlier and remained on inotersen, suggesting that treatment with inotersen altered the natural history of the disease. Importantly, no new safety concerns were identified, and there was no evidence of increased risk for grade 4 thrombocytopenia or glomerulonephritis with increased duration of inotersen exposure. Taken together, these data confirm the urgency to treat patients as early as possible.

##### Patisiran

Patisiran, a triweekly intravenously administered TTR‐directed, double‐stranded, small interfering RNA, is approved in the United States for the treatment of the polyneuropathy of hATTR in adults (Alnylam Pharmaceuticals Inc., 2018) and in Europe for the treatment of hATTR in adults with stage 1 or 2 polyneuropathy (Alnylam Netherlands B.V., 2018). It is formulated as a lipid complex to ensure adequate targeting to the liver (Table 2) (Alnylam Pharmaceuticals Inc., 2018; Suhr et al., 2015). There is the potential for infusion‐related reactions to occur because of the lipid‐based delivery of patisiran; therefore, patients receive a premedication regimen consisting of an intravenous corticosteroid (i.e., dexamethasone 10 mg or equivalent), H1 blocker, and H2 blocker, as well as oral acetaminophen (Alnylam Pharmaceuticals Inc., 2018; Suhr et al., 2015).

The phase 3, randomized, double‐blind, placebo‐controlled study APOLLO (clinicaltrials.gov identifier: NCT01960348) evaluated the efficacy and safety of patisiran compared with placebo (Adams, Gonzalez‐Duarte, O'Riordan, Yang, Ueda, et al., 2018). A total of 225 patients with hATTR polyneuropathy were randomly assigned 2:1 to patisiran 0.3 mg/kg once every 3 weeks (*n* = 148) or placebo (*n* = 77) and were stratified by NIS, disease onset/genotype, and previous stabilizer use. Baseline demographics and disease characteristics were generally balanced between treatment groups, although a slightly higher proportion of patients in the overall population had disease stage 2 (53%) than disease stage 1 (46%). Additionally, a greater proportion of patients receiving patisiran had cardiomyopathy compared with patients receiving placebo (61% vs. 47%). Patisiran treatment resulted in a median TTR reduction from baseline (measured postdose) of 81% over 18 months of treatment (Adams, Gonzalez‐Duarte, O'Riordan, Yang, Ueda, et al., 2018).

In patients receiving patisiran, a statistically significant improvement in mNIS + 7 (primary end point) was achieved at 18 months (*p* ˂ .001) compared with placebo, with effects seen as early as 9 months (Adams, Gonzalez‐Duarte, O'Riordan, Yang, Ueda, et al., 2018). A similar effect was observed for mNIS + 7 across all subgroups based on study stratification factors. Significant improvement in neuropathy‐related QOL, as indicated by Norfolk QOL‐DN (secondary end point), was also observed at 18 months (*p* ˂ .001) with patisiran compared with placebo (Adams, Gonzalez‐Duarte, O'Riordan, Yang, Ueda, et al., 2018). Similar to mNIS + 7, a benefit in favor of patisiran was observed for Norfolk QOL‐DN across all subgroups, indicating a broad clinical benefit. In addition to improvement in neuropathy and QOL, improvement from baseline in favor of patisiran was observed at 18 months for gait speed (measured by 10‐m walk test), nutritional status (measured by mBMI), and autonomic symptoms (measured by Composite Autonomic Symptom Score 31) (Adams, Gonzalez‐Duarte, O'Riordan, Yang, Ueda, et al., 2018). In the subset of patients with cardiomyopathy, patisiran was associated with significant improvements for the exploratory end points, mean left ventricular wall thickness (*p* = .02) and longitudinal strain (*p* = .02), compared with placebo at 18 months (Adams, Gonzalez‐Duarte, O'Riordan, Yang, Ueda, et al., 2018).

In addition to Norfolk QOL‐DN, QOL was also assessed using Rasch‐built Overall Disability Scale (R‐ODS)—a measure of patient‐perceived activity and social participation limitations—and EuroQOL‐5 dimension‐5 level (EQ‐5D‐5L) and EuroQOL‐visual analog scale (EQ‐VAS), two measures of patient‐perceived overall health status (van Nes et al., 2011; van Reenan & Jannsen, 2015; Regnault et al., 2017). Although R‐ODS was originally developed for patients with other neuropathies (e.g., Guillain–Barré syndrome), it has purported applicability to patients with hATTR (Regnault et al., 2017); significant differences in R‐ODS were observed with patisiran compared with placebo at 18 months (Adams, Gonzalez‐Duarte, O'Riordan, Yang, Ueda, et al., 2018). Additionally, significant differences in EQ‐5D‐5L and EQ‐VAS were observed with patisiran compared with placebo at 18 months (Adams, Gonzalez‐Duarte, O'Riordan, Yang, Yamashita, et al. 2018).

Common adverse events that occurred more frequently in patients receiving patisiran included peripheral edema, upper respiratory tract infections, and infusion‐related reactions (Adams, Gonzalez‐Duarte, O'Riordan, Yang, Ueda, et al., 2018; Alnylam Pharmaceuticals Inc., 2018). Infusion‐related reaction symptoms included back pain, flushing, nausea, abdominal pain, headache, arthralgia, and dyspnea. Seven (5%) patients experienced an infusion‐related reaction that led to treatment interruption. A similar proportion of severe and serious adverse events occurred in patients receiving patisiran and placebo, with approximately one‐third of patients experiencing either type of adverse event. Notably, more patients receiving patisiran than placebo experienced a serious adverse event of complete atrioventricular block. Seven (5%) deaths (all cardiac‐related) occurred in patients receiving patisiran, and six (8%) occurred in patients receiving placebo (none cardiac‐related).

The sustained activity of patisiran was observed with extended dosing in an ongoing global open‐label extension study (clinicaltrials.gov identifier: NCT02510261) (González‐Duarte et al., 2018; Suhr et al., 2018). In APOLLO, 86% of patients completed the 18‐month treatment period, and ˃95% of patients were eligible for participation in the open‐label extension study (Adams, Gonzalez‐Duarte, O'Riordan, Yang, Ueda, et al., 2018). Treatment with patisiran for up to 29 months in patients who continued to receive patisiran in the extension study demonstrated disease stabilization, as evidenced by mNIS + 7 (González‐Duarte et al., 2018). Disease stabilization was also observed with patisiran in patients who previously received placebo (González‐Duarte et al., 2018). No new safety concerns were identified in the extension study (González‐Duarte et al., 2018).

### 3.4 Emerging/investigational therapies

Several investigational therapies are being evaluated for the treatment of hATTR amyloidosis. These therapies target specific aspects of the amyloidogenic cascade and, in some cases, may offer advantages over existing agents. Several agents that stabilize TTR are being investigated, including epigallocatechin‐3‐gallate (EGCG), AG‐10, and CHF5074. EGCG, a catechin in green tea, has demonstrated increased stabilization of TTR tetramers and a reduction in TTR deposition (Ferreira, Saraiva, & Almeida, 2012). In a mouse model of hATTR amyloidosis with peripheral nervous system involvement, EGCG 100 mg kg^−1^ day^−1^ for 6 weeks produced a significant reduction in TTR deposition in dorsal root ganglia and sciatic nerve (Ferreira et al., 2012). AG‐10, a kinetic stabilizer, binds with high affinity and negative cooperativity to TTR (Penchala et al., 2013). In preclinical studies, AG‐10 stabilizes tetramers composed of wild‐type and mutated TTR equally well, whereas tafamidis showed greater stabilization of wild‐type than mutated TTR (Penchala et al., 2013). CHF5074, an NSAID derivative without cyclooxygenase inhibitory properties, is another molecule that has demonstrated stabilization of wild‐type and mutant TTR tetramers (Mu et al., 2015). The absence of cyclooxygenase inhibitory activity offers an advantage over other NSAID‐based TTR stabilizers in that it may prevent unwanted adverse effects associated with NSAIDs. Additional testing is needed to confirm whether that is the case for CHF5074. Monoclonal antibodies directed at misfolded TTR are another therapeutic strategy being investigated for ATTR amyloidosis. Preclinical activity demonstrated the ability of monoclonal antibodies to prevent amyloid fibril formation (Higaki et al., 2016). Additionally, antibody‐dependent phagocytic uptake of misfolded TTR was observed (Higaki et al., 2016). PRX004 is an investigational antibody being evaluated in a phase 1, open‐label, dose‐escalation study (clinicaltrials.gov identifier: NCT03336580) (US National Library of Medicine, 2018b). Lastly, second‐generation *TTR* gene knockdown agents are also emerging. AKCEA‐TTR‐LRx (Akcea Therapeutics, Inc., 2019), a subcutaneous antisense oligonucleotide with improved hepatic targeting with the potential for less frequent and lower doses of drug, is in phase 3 planning, and vutrisiran (ALN‐TTRsc02) (US National Library of Medicine, 2018a), a subcutaneous RNA interference therapeutic with the potential for less frequent doses of drug, will be evaluated in the phase 3 HELIOS‐A study (clinicaltrials.gov identifier: NCT03759379).

## 4 CONCLUSIONS

Until recently, there have been few treatment options for patients with hATTR. Recent results of clinical studies for tafamidis, inotersen, and patisiran represent a significant advance in the field of amyloidosis. The results of the tafamidis ATTR‐ACT study demonstrated significant reductions in death and cardiovascular hospitalization for patients with ATTR cardiomyopathy, supporting the role of stabilizer therapy in cardiac patients, especially those with wtATTR. The unique mechanisms of action of inotersen and patisiran overcome many limitations of previous therapies for patients with hATTR. Both agents are efficacious in patients with early‐ and late‐stage disease and improve, halt, or slow neuropathy progression; however, differences in formulation, dosing, requirements for premedications, and safety monitoring have important implications for patients and for selecting therapies. Importantly, direct comparison of inotersen and patisiran is not possible given differences in the NEURO‐TTR and APOLLO study designs, end points, and patient populations. Similarly, direct comparison of inotersen and patisiran with tafamidis for patients with cardiac disease is not possible given differences in the NEURO‐TTR, APOLLO, and ATTR‐ACT studies. Additional research is necessary to determine the optimal treatment for a given patient with ATTR. It is possible that gene silencing therapy in combination with TTR‐stabilizing therapy or an agent that removes fibril deposition may be beneficial. As additional evidence for current and emerging therapies expands, the outlook for patients with ATTR is becoming more promising, offering much‐needed hope for a debilitating and life‐threatening disease.

## AUTHOR CONTRIBUTIONS

All authors contributed equally to this article, take responsibility for its contents, and were involved in the planning, preparation, and review/revision of this article. All authors approved the final draft and agreed to submit the article for consideration of publication. Medical writing support was provided by ApotheCom and funded by Akcea Therapeutics. Akcea Therapeutics had no role in determining the content of the article.

## DATA AVAILABILITY STATEMENT

The data that support the findings of this study were derived from the following resource available in the public domain: PubMed at https://www.ncbi.nlm.nih.gov/pubmed.
